# Supplementary material for: Overlapping cell population expression profiling and regulatory inference in C. elegans
Source: BMC Genomics. 2016 Feb 29;17:159. doi: 10.1186/s12864-016-2482-z (PMC4772325; doi:10.1186/s12864-016-2482-z)
Supplement: Additional file 13: — Web supplement. (DOC 21 kb) [file 12864_2016_2482_MOESM13_ESM.zip › sortWeb/clusters/hier.300.clusters/287.html]

Cluster 287 

## Cluster 287

### Expression

| cnd-1 rep. 1 | cnd-1 rep. 2 | cnd-1 rep. 3 | pha-4 rep. 1 | pha-4 rep. 2 | pha-4 rep. 3 | ceh-27 | ceh-36 | ceh-6 | F21D5.9 | mir-57 | mls-2 | pal-1 | pros-1 | ttx-3 | unc-130 | hlh-16 | irx-1 | ceh-6 (+) hlh-16 (+) | ceh-6 (+) hlh-16 (-) | ceh-6 (-) hlh-16 (+) | cnd-1 singlets | pha-4 singlets | 0 | 60 | 120 | 150 | 180 | 240 | 330 | 390 | 420 | 480 | 540 | 570 | 600 | 630 | 660 | NAME | Functional description |
| --- | --- | --- | --- | --- | --- | --- | --- | --- | --- | --- | --- | --- | --- | --- | --- | --- | --- | --- | --- | --- | --- | --- | --- | --- | --- | --- | --- | --- | --- | --- | --- | --- | --- | --- | --- | --- | --- | --- | --- |
|  |  |  |  |  |  |  |  |  |  |  |  |  |  |  |  |  |  |  |  |  |  |  |  |  |  |  |  |  |  |  |  |  |  |  |  |  |  | ZC581.2 |  |
|  |  |  |  |  |  |  |  |  |  |  |  |  |  |  |  |  |  |  |  |  |  |  |  |  |  |  |  |  |  |  |  |  |  |  |  |  |  | F45D3.1 |  |
|  |  |  |  |  |  |  |  |  |  |  |  |  |  |  |  |  |  |  |  |  |  |  |  |  |  |  |  |  |  |  |  |  |  |  |  |  |  | F33C8.2 |  |
|  |  |  |  |  |  |  |  |  |  |  |  |  |  |  |  |  |  |  |  |  |  |  |  |  |  |  |  |  |  |  |  |  |  |  |  |  |  | *lgc-36* | Ligand-Gated ion Channel |
|  |  |  |  |  |  |  |  |  |  |  |  |  |  |  |  |  |  |  |  |  |  |  |  |  |  |  |  |  |  |  |  |  |  |  |  |  |  | F46C8.1 |  |
|  |  |  |  |  |  |  |  |  |  |  |  |  |  |  |  |  |  |  |  |  |  |  |  |  |  |  |  |  |  |  |  |  |  |  |  |  |  | T04C10.3 |  |
|  |  |  |  |  |  |  |  |  |  |  |  |  |  |  |  |  |  |  |  |  |  |  |  |  |  |  |  |  |  |  |  |  |  |  |  |  |  | ZK938.3 |  |
|  |  |  |  |  |  |  |  |  |  |  |  |  |  |  |  |  |  |  |  |  |  |  |  |  |  |  |  |  |  |  |  |  |  |  |  |  |  | R09E10.1 |  |
|  |  |  |  |  |  |  |  |  |  |  |  |  |  |  |  |  |  |  |  |  |  |  |  |  |  |  |  |  |  |  |  |  |  |  |  |  |  | K11C4.1 |  |
|  |  |  |  |  |  |  |  |  |  |  |  |  |  |  |  |  |  |  |  |  |  |  |  |  |  |  |  |  |  |  |  |  |  |  |  |  |  | *zig-8* | 2 (Zwei) IG domain protein |
|  |  |  |  |  |  |  |  |  |  |  |  |  |  |  |  |  |  |  |  |  |  |  |  |  |  |  |  |  |  |  |  |  |  |  |  |  |  | *frpr-3* | FMRFamide Peptide Receptor family |
|  |  |  |  |  |  |  |  |  |  |  |  |  |  |  |  |  |  |  |  |  |  |  |  |  |  |  |  |  |  |  |  |  |  |  |  |  |  | *dmsr-9* | DroMyoSuppressin Receptor related |
|  |  |  |  |  |  |  |  |  |  |  |  |  |  |  |  |  |  |  |  |  |  |  |  |  |  |  |  |  |  |  |  |  |  |  |  |  |  | R11D1.7 |  |
|  |  |  |  |  |  |  |  |  |  |  |  |  |  |  |  |  |  |  |  |  |  |  |  |  |  |  |  |  |  |  |  |  |  |  |  |  |  | Y11D7A.8 |  |
|  |  |  |  |  |  |  |  |  |  |  |  |  |  |  |  |  |  |  |  |  |  |  |  |  |  |  |  |  |  |  |  |  |  |  |  |  |  | *sri-72* | Serpentine Receptor, class I |
|  |  |  |  |  |  |  |  |  |  |  |  |  |  |  |  |  |  |  |  |  |  |  |  |  |  |  |  |  |  |  |  |  |  |  |  |  |  | F22F1.3 |  |
|  |  |  |  |  |  |  |  |  |  |  |  |  |  |  |  |  |  |  |  |  |  |  |  |  |  |  |  |  |  |  |  |  |  |  |  |  |  | Y95B8A.12 |  |
|  |  |  |  |  |  |  |  |  |  |  |  |  |  |  |  |  |  |  |  |  |  |  |  |  |  |  |  |  |  |  |  |  |  |  |  |  |  | *scd-2* | Suppressor of Constitutive Dauer formation |
|  |  |  |  |  |  |  |  |  |  |  |  |  |  |  |  |  |  |  |  |  |  |  |  |  |  |  |  |  |  |  |  |  |  |  |  |  |  | *nlp-38* | Neuropeptide-Like Protein |
|  |  |  |  |  |  |  |  |  |  |  |  |  |  |  |  |  |  |  |  |  |  |  |  |  |  |  |  |  |  |  |  |  |  |  |  |  |  | *ins-18* | INSulin related |
|  |  |  |  |  |  |  |  |  |  |  |  |  |  |  |  |  |  |  |  |  |  |  |  |  |  |  |  |  |  |  |  |  |  |  |  |  |  | *flp-19* | FMRF-Like Peptide |
|  |  |  |  |  |  |  |  |  |  |  |  |  |  |  |  |  |  |  |  |  |  |  |  |  |  |  |  |  |  |  |  |  |  |  |  |  |  | F09E5.16 |  |
|  |  |  |  |  |  |  |  |  |  |  |  |  |  |  |  |  |  |  |  |  |  |  |  |  |  |  |  |  |  |  |  |  |  |  |  |  |  | F41H10.2 |  |
|  |  |  |  |  |  |  |  |  |  |  |  |  |  |  |  |  |  |  |  |  |  |  |  |  |  |  |  |  |  |  |  |  |  |  |  |  |  | T23F2.4 |  |
|  |  |  |  |  |  |  |  |  |  |  |  |  |  |  |  |  |  |  |  |  |  |  |  |  |  |  |  |  |  |  |  |  |  |  |  |  |  | *gnrr-7* | human GoNadotropin-Releasing hormone Receptor (GnRHR) related |
|  |  |  |  |  |  |  |  |  |  |  |  |  |  |  |  |  |  |  |  |  |  |  |  |  |  |  |  |  |  |  |  |  |  |  |  |  |  | C47E8.3 |  |
|  |  |  |  |  |  |  |  |  |  |  |  |  |  |  |  |  |  |  |  |  |  |  |  |  |  |  |  |  |  |  |  |  |  |  |  |  |  | F35H10.2 |  |
|  |  |  |  |  |  |  |  |  |  |  |  |  |  |  |  |  |  |  |  |  |  |  |  |  |  |  |  |  |  |  |  |  |  |  |  |  |  | *bath-37* | BTB and MATH domain containing |
|  |  |  |  |  |  |  |  |  |  |  |  |  |  |  |  |  |  |  |  |  |  |  |  |  |  |  |  |  |  |  |  |  |  |  |  |  |  | F55F1.1 |  |
|  |  |  |  |  |  |  |  |  |  |  |  |  |  |  |  |  |  |  |  |  |  |  |  |  |  |  |  |  |  |  |  |  |  |  |  |  |  | C18F3.11 |  |
|  |  |  |  |  |  |  |  |  |  |  |  |  |  |  |  |  |  |  |  |  |  |  |  |  |  |  |  |  |  |  |  |  |  |  |  |  |  | *fbxa-44* | F-box A protein |
|  |  |  |  |  |  |  |  |  |  |  |  |  |  |  |  |  |  |  |  |  |  |  |  |  |  |  |  |  |  |  |  |  |  |  |  |  |  | *srz-82* | Serpentine Receptor, class Z |
|  |  |  |  |  |  |  |  |  |  |  |  |  |  |  |  |  |  |  |  |  |  |  |  |  |  |  |  |  |  |  |  |  |  |  |  |  |  | F13E9.11 |  |
|  |  |  |  |  |  |  |  |  |  |  |  |  |  |  |  |  |  |  |  |  |  |  |  |  |  |  |  |  |  |  |  |  |  |  |  |  |  | *fbxa-155* | F-box A protein |
|  |  |  |  |  |  |  |  |  |  |  |  |  |  |  |  |  |  |  |  |  |  |  |  |  |  |  |  |  |  |  |  |  |  |  |  |  |  | ZK697.3 |  |
|  |  |  |  |  |  |  |  |  |  |  |  |  |  |  |  |  |  |  |  |  |  |  |  |  |  |  |  |  |  |  |  |  |  |  |  |  |  | F08G2.8 |  |
|  |  |  |  |  |  |  |  |  |  |  |  |  |  |  |  |  |  |  |  |  |  |  |  |  |  |  |  |  |  |  |  |  |  |  |  |  |  | cTel3X.3 |  |
|  |  |  |  |  |  |  |  |  |  |  |  |  |  |  |  |  |  |  |  |  |  |  |  |  |  |  |  |  |  |  |  |  |  |  |  |  |  | *nlp-41* | Neuropeptide-Like Protein |
|  |  |  |  |  |  |  |  |  |  |  |  |  |  |  |  |  |  |  |  |  |  |  |  |  |  |  |  |  |  |  |  |  |  |  |  |  |  | D2096.9 |  |
|  |  |  |  |  |  |  |  |  |  |  |  |  |  |  |  |  |  |  |  |  |  |  |  |  |  |  |  |  |  |  |  |  |  |  |  |  |  | F56H11.8 |  |
|  |  |  |  |  |  |  |  |  |  |  |  |  |  |  |  |  |  |  |  |  |  |  |  |  |  |  |  |  |  |  |  |  |  |  |  |  |  | T04C12.9 |  |
|  |  |  |  |  |  |  |  |  |  |  |  |  |  |  |  |  |  |  |  |  |  |  |  |  |  |  |  |  |  |  |  |  |  |  |  |  |  | *flp-6* | FMRF-Like Peptide |
|  |  |  |  |  |  |  |  |  |  |  |  |  |  |  |  |  |  |  |  |  |  |  |  |  |  |  |  |  |  |  |  |  |  |  |  |  |  | K10C8.4 |  |
|  |  |  |  |  |  |  |  |  |  |  |  |  |  |  |  |  |  |  |  |  |  |  |  |  |  |  |  |  |  |  |  |  |  |  |  |  |  | T12E12.44 |  |
|  |  |  |  |  |  |  |  |  |  |  |  |  |  |  |  |  |  |  |  |  |  |  |  |  |  |  |  |  |  |  |  |  |  |  |  |  |  | C24D10.9 |  |
|  |  |  |  |  |  |  |  |  |  |  |  |  |  |  |  |  |  |  |  |  |  |  |  |  |  |  |  |  |  |  |  |  |  |  |  |  |  | *rgef-1* | Rap Guanine nucleotide Exchange Factor homolog |
|  |  |  |  |  |  |  |  |  |  |  |  |  |  |  |  |  |  |  |  |  |  |  |  |  |  |  |  |  |  |  |  |  |  |  |  |  |  | F38E9.5 |  |
|  |  |  |  |  |  |  |  |  |  |  |  |  |  |  |  |  |  |  |  |  |  |  |  |  |  |  |  |  |  |  |  |  |  |  |  |  |  | *crtc-1* | CREB-Regulated Transcription Coactivator homolog |
|  |  |  |  |  |  |  |  |  |  |  |  |  |  |  |  |  |  |  |  |  |  |  |  |  |  |  |  |  |  |  |  |  |  |  |  |  |  | *dlk-1* | Dual-Leucine zipper Kinase |
|  |  |  |  |  |  |  |  |  |  |  |  |  |  |  |  |  |  |  |  |  |  |  |  |  |  |  |  |  |  |  |  |  |  |  |  |  |  | *ctn-1* | alpha-CaTuliN (catenin/vinculin related) |
|  |  |  |  |  |  |  |  |  |  |  |  |  |  |  |  |  |  |  |  |  |  |  |  |  |  |  |  |  |  |  |  |  |  |  |  |  |  | ZC132.9 |  |
|  |  |  |  |  |  |  |  |  |  |  |  |  |  |  |  |  |  |  |  |  |  |  |  |  |  |  |  |  |  |  |  |  |  |  |  |  |  | *nhr-281* | Nuclear Hormone Receptor family |
|  |  |  |  |  |  |  |  |  |  |  |  |  |  |  |  |  |  |  |  |  |  |  |  |  |  |  |  |  |  |  |  |  |  |  |  |  |  | F31A3.3 |  |
|  |  |  |  |  |  |  |  |  |  |  |  |  |  |  |  |  |  |  |  |  |  |  |  |  |  |  |  |  |  |  |  |  |  |  |  |  |  | R06C1.6 |  |
|  |  |  |  |  |  |  |  |  |  |  |  |  |  |  |  |  |  |  |  |  |  |  |  |  |  |  |  |  |  |  |  |  |  |  |  |  |  | F02E11.7 |  |
|  |  |  |  |  |  |  |  |  |  |  |  |  |  |  |  |  |  |  |  |  |  |  |  |  |  |  |  |  |  |  |  |  |  |  |  |  |  | *srh-166* | Serpentine Receptor, class H |
|  |  |  |  |  |  |  |  |  |  |  |  |  |  |  |  |  |  |  |  |  |  |  |  |  |  |  |  |  |  |  |  |  |  |  |  |  |  | F47B10.3 |  |
|  |  |  |  |  |  |  |  |  |  |  |  |  |  |  |  |  |  |  |  |  |  |  |  |  |  |  |  |  |  |  |  |  |  |  |  |  |  | *lgc-30* | Ligand-Gated ion Channel |
|  |  |  |  |  |  |  |  |  |  |  |  |  |  |  |  |  |  |  |  |  |  |  |  |  |  |  |  |  |  |  |  |  |  |  |  |  |  | F08F3.1 |  |
|  |  |  |  |  |  |  |  |  |  |  |  |  |  |  |  |  |  |  |  |  |  |  |  |  |  |  |  |  |  |  |  |  |  |  |  |  |  | *glb-16* | GLoBin related |
|  |  |  |  |  |  |  |  |  |  |  |  |  |  |  |  |  |  |  |  |  |  |  |  |  |  |  |  |  |  |  |  |  |  |  |  |  |  | C06A6.7 |  |
|  |  |  |  |  |  |  |  |  |  |  |  |  |  |  |  |  |  |  |  |  |  |  |  |  |  |  |  |  |  |  |  |  |  |  |  |  |  | *gcy-33* | Guanylyl CYclase |
|  |  |  |  |  |  |  |  |  |  |  |  |  |  |  |  |  |  |  |  |  |  |  |  |  |  |  |  |  |  |  |  |  |  |  |  |  |  | H10E21.5 |  |
|  |  |  |  |  |  |  |  |  |  |  |  |  |  |  |  |  |  |  |  |  |  |  |  |  |  |  |  |  |  |  |  |  |  |  |  |  |  | *mec-12* | MEChanosensory abnormality |
|  |  |  |  |  |  |  |  |  |  |  |  |  |  |  |  |  |  |  |  |  |  |  |  |  |  |  |  |  |  |  |  |  |  |  |  |  |  | C03F11.4 |  |
|  |  |  |  |  |  |  |  |  |  |  |  |  |  |  |  |  |  |  |  |  |  |  |  |  |  |  |  |  |  |  |  |  |  |  |  |  |  | *irk-3* | Inward Rectifying K (potassium) channel family |
|  |  |  |  |  |  |  |  |  |  |  |  |  |  |  |  |  |  |  |  |  |  |  |  |  |  |  |  |  |  |  |  |  |  |  |  |  |  | *flp-16* | FMRF-Like Peptide |
|  |  |  |  |  |  |  |  |  |  |  |  |  |  |  |  |  |  |  |  |  |  |  |  |  |  |  |  |  |  |  |  |  |  |  |  |  |  | *hsp-110* | Heat Shock Protein |
|  |  |  |  |  |  |  |  |  |  |  |  |  |  |  |  |  |  |  |  |  |  |  |  |  |  |  |  |  |  |  |  |  |  |  |  |  |  | R10F2.4 |  |
|  |  |  |  |  |  |  |  |  |  |  |  |  |  |  |  |  |  |  |  |  |  |  |  |  |  |  |  |  |  |  |  |  |  |  |  |  |  | K06H7.1 |  |
|  |  |  |  |  |  |  |  |  |  |  |  |  |  |  |  |  |  |  |  |  |  |  |  |  |  |  |  |  |  |  |  |  |  |  |  |  |  | *shl-1* | SHaL family of potassium channels |
|  |  |  |  |  |  |  |  |  |  |  |  |  |  |  |  |  |  |  |  |  |  |  |  |  |  |  |  |  |  |  |  |  |  |  |  |  |  | *dmsr-1* | DroMyoSuppressin Receptor related |
|  |  |  |  |  |  |  |  |  |  |  |  |  |  |  |  |  |  |  |  |  |  |  |  |  |  |  |  |  |  |  |  |  |  |  |  |  |  | *egl-13* | EGg Laying defective |
|  |  |  |  |  |  |  |  |  |  |  |  |  |  |  |  |  |  |  |  |  |  |  |  |  |  |  |  |  |  |  |  |  |  |  |  |  |  | *dkf-2* | D Kinase Family |
|  |  |  |  |  |  |  |  |  |  |  |  |  |  |  |  |  |  |  |  |  |  |  |  |  |  |  |  |  |  |  |  |  |  |  |  |  |  | *linc-56* | Long Intervening Non-Coding RNA |
|  |  |  |  |  |  |  |  |  |  |  |  |  |  |  |  |  |  |  |  |  |  |  |  |  |  |  |  |  |  |  |  |  |  |  |  |  |  | *tbc-8* | TBC (Tre-2/Bub2/Cdc16) domain family |
|  |  |  |  |  |  |  |  |  |  |  |  |  |  |  |  |  |  |  |  |  |  |  |  |  |  |  |  |  |  |  |  |  |  |  |  |  |  | *sipa-1* | SIPA (vertebrate Signal-Induced Proliferation-Associated) homolog |
|  |  |  |  |  |  |  |  |  |  |  |  |  |  |  |  |  |  |  |  |  |  |  |  |  |  |  |  |  |  |  |  |  |  |  |  |  |  | *rbf-1* | RaBPHilin |
|  |  |  |  |  |  |  |  |  |  |  |  |  |  |  |  |  |  |  |  |  |  |  |  |  |  |  |  |  |  |  |  |  |  |  |  |  |  | *pdfr-1* | PDF Receptor homolog |
|  |  |  |  |  |  |  |  |  |  |  |  |  |  |  |  |  |  |  |  |  |  |  |  |  |  |  |  |  |  |  |  |  |  |  |  |  |  | *kcnl-2* | KCNN (potassium K ChaNNel, calcium activated)-Like |
|  |  |  |  |  |  |  |  |  |  |  |  |  |  |  |  |  |  |  |  |  |  |  |  |  |  |  |  |  |  |  |  |  |  |  |  |  |  | *tyra-3* | TYRAmine receptor |

### Phenotypes enriched

none found

### Anatomy terms enriched

none found

### GO terms enriched

|  |  |  |
| --- | --- | --- |
| **GO term** | **Number of genes** | **FDR-corrected p-value** |
| signaling | 13 | 0.0044 |
| response to stimulus | 15 | 0.0140 |
| signal transduction | 10 | 0.0170 |
| neuropeptide signaling pathway | 3 | 0.0340 |
| G-protein coupled receptor activity | 5 | 0.0380 |

### Expression clusters enriched

|  |  |  |  |
| --- | --- | --- | --- |
| **Group name** | **Number in cluster** | **Enrichment** | **FDR corrected p** |
| Larval Pan-neural Enriched Genes. | 31 | 3.88 | 6.13e-09 |
| Genes significantly enriched (> 2x, FDR < 5%) in a particular cell-type versus a reference sample of all cells at the same stage. WBPaper00037950:all-neurons\_larva\_enriched | 24 | 4.59 | 9.70e-08 |
| Genes significantly enriched (> 2x, FDR < 5%) in a particular cell-type versus a reference sample of all cells at the same stage. WBPaper00037950:glr-1(+)-neurons\_larva\_enriched | 19 | 5.57 | 4.73e-07 |
| WT-Pico Pan-neural Enriched Genes, with genes found multiple times in a single dataset removed (without dups). | 27 | 3.26 | 7.43e-06 |
| Genes significantly enriched (> 2x, FDR < 5%) in a particular cell-type versus a reference sample of all cells at the same stage. WBPaper00037950:A-class-motor-neurons\_larva\_enriched | 16 | 4.50 | 1.57e-04 |
| Genes that show selective expression in a subset of cell types vs broadly expressed in many cell types. Correspond to 20% - 57% of enriched\_genes for a given cell type. WBPaper00037950:all-neurons\_larva\_SelectivelyEnriched | 12 | 4.26 | 6.34e-03 |
| Genes that show selective expression in a subset of cell types vs broadly expressed in many cell types. Correspond to 20% - 57% of enriched\_genes for a given cell type. WBPaper00037950:BAG-neuron\_embryo\_SelectivelyEnriched | 7 | 6.48 | 2.44e-02 |
| Genes enriched in neuronal miRNA-induced silencing complexes (miRISC) as detected by immunoprecipitations and microarray analysis. | 12 | 3.36 | 4.63e-02 |

### Motifs enriched

|  |  |  |  |  |  |
| --- | --- | --- | --- | --- | --- |
| **Motif** | **Logo** | **Possible orthologs** | **Number of motifs in cluster** | **Enrichment** | **FDR corrected p** |
| nau\_da\_SANGER\_5\_FBgn0002922 |  | hlh-15 K02D7.2 hlh-2 hlh-1 | 49 | 1.95 | 1.6e-05 |
| Ap\_Cell\_FBgn0000099 |  | mls-2 (0.61) ceh-23 (0.57) ceh-18 (0.57) ceh-36 alr-1 lim-7 lim-4 ceh-10 ceh-31 lin-39 ceh-14 ceh-12 ceh-30 ceh-2 pha-2 ceh-53 ceh-16 lim-6 ceh-45 dsc-1 and 7 others  [full list] | 60 | 1.68 | 1.8e-05 |
| V$SP1\_Q6 |  | ZC328.2 klf-2 klf-1 | 47 | 2.00 | 2.1e-05 |
| POU4F3\_1 |  | ceh-18 (0.57) unc-86 | 61 | 1.64 | 2.8e-05 |
| luna\_SANGER\_5\_FBgn0040765 |  | klf-2 klf-1 | 33 | 2.56 | 3.0e-05 |
| Sp4\_1011 |  | sptf-3 klf-2 klf-1 | 35 | 2.42 | 3.7e-05 |
| ISL1\_f1 |  | lim-7 lin-39 cfi-1 unc-86 | 45 | 1.98 | 6.0e-05 |
| POU3F3\_1 |  | sox-4 (0.74) ceh-18 (0.57) ceh-6 tbp-1 | 64 | 1.55 | 6.1e-05 |
| ESRRA\_2 |  | nhr-71 (0.59) nhr-6 nhr-2 nhr-213 nhr-10 nhr-68 | 77 | 1.31 | 6.4e-05 |
| pTH5005 |  | crh-1 W08E12.1 attf-1 | 53 | 1.75 | 8.2e-05 |
| Smad3\_3805 |  | daf-8 | 49 | 1.83 | 1.1e-04 |
| pTH4998 |  | ZC328.2 klf-2 klf-1 | 44 | 1.96 | 1.2e-04 |
| pTH5080 |  | crh-1 fos-1 atf-5 | 35 | 2.25 | 1.7e-04 |
| gl\_FlyReg\_FBgn0004618 |  | ceh-24 (0.59) ces-1 C34H4.5 | 55 | 1.67 | 1.9e-04 |
| V$AREB6\_01 |  | tbx-39 ztf-6 | 49 | 1.78 | 2.3e-04 |
| MA0165.1 |  | ceh-24 (0.59) pal-1 php-3 lin-39 | 74 | 1.33 | 2.4e-04 |
| K562\_SP2\_HudsonAlpha |  | klf-2 | 70 | 1.40 | 2.6e-04 |
| V$CDXA\_01 |  | php-3 ceh-13 | 69 | 1.41 | 2.7e-04 |
| Atf6\_SANGER\_5\_FBgn0033010 |  | atf-6 crh-1 fos-1 atf-7 | 77 | 1.27 | 3.3e-04 |
| V$FOXO4\_01 |  | fkh-10 daf-16 let-381 pha-4 lin-31 fkh-7 | 77 | 1.27 | 3.3e-04 |
| V$SRF\_Q6 |  | hlh-10 unc-120 | 46 | 1.83 | 3.4e-04 |
| MA0174.1 |  | ceh-24 (0.59) pal-1 lin-39 | 71 | 1.37 | 3.5e-04 |
| MEIS3\_1 |  | ceh-32 | 59 | 1.56 | 3.6e-04 |
| FOXF1\_f1 |  | let-381 lin-31 | 60 | 1.54 | 3.8e-04 |
| Dll\_SOLEXA\_FBgn0000157 |  | ceh-18 (0.57) alr-1 npax-3 lin-39 ceh-12 ceh-53 ceh-45 ceh-1 ceh-43 | 77 | 1.27 | 3.9e-04 |
| MAFA\_f1 |  | F45H11.6 | 53 | 1.67 | 4.0e-04 |
| MA0188.1 |  | ceh-8 (0.7) ceh-24 (0.59) alr-1 lim-7 ceh-9 ceh-31 lin-39 ceh-30 ceh-1 ceh-43 | 77 | 1.27 | 4.1e-04 |
| HXD10\_f1 |  | php-3 nhr-2 | 58 | 1.57 | 4.1e-04 |
| SMAD3\_1 |  | sma-4 hlh-8 daf-8 | 39 | 2.00 | 4.7e-04 |
| pTH9326 |  | nhr-122 | 59 | 1.55 | 4.8e-04 |
| NKX22\_si |  | ceh-24 (0.59) ceh-22 dsc-1 | 78 | 1.24 | 5.1e-04 |
| V$CETS1P54\_02 |  | C52B9.2 | 62 | 1.49 | 6.3e-04 |
| MA0485.1 |  | php-3 lin-39 | 40 | 1.94 | 6.5e-04 |
| pTH3997 |  | C04F5.9 | 67 | 1.41 | 6.8e-04 |
| MA0227.1 |  | ceh-32 | 75 | 1.29 | 7.8e-04 |
| CART1\_1 |  | alr-1 ceh-14 | 53 | 1.63 | 8.0e-04 |
| tup\_SOLEXA\_10\_FBgn0003896 |  | lim-7 ceh-9 ceh-31 ceh-16 ceh-1 ceh-43 | 77 | 1.25 | 8.1e-04 |
| Lhx1\_2240 |  | lim-7 | 79 | 1.21 | 9.0e-04 |
| pTH5812 |  | ceh-14 | 46 | 1.76 | 9.0e-04 |
| Pou3f2\_2824 |  | ceh-18 (0.57) lin-39 ceh-6 | 78 | 1.23 | 9.3e-04 |
| Gmeb1\_1745 |  | attf-1 | 12 | 4.97 | 9.5e-04 |
| ELF1\_1 |  | nhr-19 lin-1 C24A1.2 | 61 | 1.48 | 9.9e-04 |
| Dlx3\_1030 |  | ceh-43 | 78 | 1.23 | 1.1e-03 |
| tgo\_tai\_SANGER\_5\_FBgn0015014 |  | aha-1 hlh-30 | 66 | 1.41 | 1.1e-03 |
| Hoxd1\_3448 |  | ceh-12 | 52 | 1.63 | 1.1e-03 |
| pTH8991 |  | cey-3 | 19 | 3.15 | 1.1e-03 |
| Pou2f3\_3986 |  | ceh-18 (0.57) | 56 | 1.56 | 1.1e-03 |
| pTH8745 |  | attf-1 | 12 | 4.87 | 1.1e-03 |
| pTH3819 |  | ceh-18 (0.57) | 60 | 1.49 | 1.1e-03 |
| Foxj1\_3125 |  | fkh-8 (0.6) fkh-10 daf-16 let-381 lin-31 fkh-7 | 78 | 1.23 | 1.1e-03 |
| MCR\_f1 |  | nhr-255 | 57 | 1.54 | 1.2e-03 |
| MA0150.2 |  | jun-1 (0.6) crh-1 sknr-1 F45H11.6 | 73 | 1.30 | 1.2e-03 |
| Dlx2\_2273 |  | ceh-43 | 78 | 1.22 | 1.2e-03 |
| pTH10623 |  | scrt-1 | 24 | 2.61 | 1.2e-03 |
| pTH9214 |  | cfi-1 | 56 | 1.55 | 1.3e-03 |
| V$DELTAEF1\_01 |  | hlh-2 ztf-6 | 49 | 1.67 | 1.3e-03 |
| pTH9052 |  | ces-2 C01B12.2 | 53 | 1.59 | 1.4e-03 |
| Nkx1-2\_3214 |  | ceh-30 | 47 | 1.71 | 1.4e-03 |
| pTH9216 |  | ceh-18 (0.57) | 54 | 1.58 | 1.4e-03 |
| MA0508.1 |  | blmp-1 | 47 | 1.70 | 1.5e-03 |
| Tcf1\_2666 |  | hmbx-1 | 70 | 1.34 | 1.5e-03 |
| pTH10699 |  | alr-1 ceh-53 ceh-45 dve-1 | 51 | 1.63 | 1.5e-03 |
| RORG\_f1 |  | nhr-213 nhr-118 | 56 | 1.54 | 1.6e-03 |
| pTH4325 |  | ceh-18 (0.57) | 67 | 1.38 | 1.6e-03 |
| pTH5778 |  | egl-5 | 57 | 1.52 | 1.6e-03 |
| Lbx2\_3869 |  | mls-2 (0.61) | 49 | 1.66 | 1.6e-03 |
| pTH9957 |  | irx-1 fkh-9 | 64 | 1.42 | 1.6e-03 |
| Dlx1\_1741 |  | ceh-43 | 78 | 1.22 | 1.6e-03 |
| Cutl1\_3494 |  | ceh-44 | 69 | 1.35 | 1.6e-03 |
| pTH5891 |  | nhr-49 (0.51) | 69 | 1.35 | 1.7e-03 |
| pTH9254 |  | mel-28 | 75 | 1.27 | 1.7e-03 |
| pTH10779 |  | nhr-182 | 35 | 2.00 | 1.7e-03 |
| pTH6449 |  | ceh-43 | 49 | 1.65 | 1.8e-03 |
| pTH3064 |  | crh-1 | 43 | 1.78 | 1.8e-03 |
| ETV2\_1 |  | lin-1 C24A1.2 | 64 | 1.41 | 1.8e-03 |
| pTH10811 |  | nhr-216 (0.62) | 41 | 1.82 | 1.9e-03 |
| pTH10795 |  | ZC328.2 pax-3 ref-2 ztf-14 | 43 | 1.77 | 2.0e-03 |
| pTH5808 |  | ceh-24 (0.59) pal-1 | 65 | 1.40 | 2.0e-03 |
| HXB8\_do |  | lin-39 | 50 | 1.62 | 2.1e-03 |
| MYF6\_f1 |  | hlh-15 hlh-1 | 45 | 1.72 | 2.1e-03 |
| SMAD3\_f1 |  | daf-8 | 47 | 1.68 | 2.1e-03 |
| Prrx1\_3442 |  | alr-1 ceh-10 lin-39 ceh-45 eyg-1 ceh-1 | 78 | 1.21 | 2.1e-03 |
| GATA3\_si |  | elt-1 | 43 | 1.76 | 2.2e-03 |
| PROX1\_1 |  | crh-1 ceh-26 | 70 | 1.33 | 2.3e-03 |
| NR2F1\_3 |  | nhr-15 nhr-2 | 67 | 1.36 | 2.6e-03 |
| FOXO1\_3 |  | ZC328.2 daf-16 | 55 | 1.53 | 2.6e-03 |
| MA0007.2 |  | nhr-255 npax-1 | 75 | 1.25 | 2.7e-03 |
| Hoxd13\_2356 |  | pal-1 | 45 | 1.70 | 2.8e-03 |
| V$ROAZ\_01 |  | D1081.8 C09F5.3 | 57 | 1.49 | 2.8e-03 |
| MA0482.1 |  | elt-1 ztf-29 | 80 | 1.17 | 2.8e-03 |
| NR2F1\_2 |  | nhr-2 nhr-62 | 72 | 1.29 | 2.9e-03 |
| MA0066.1 |  | nhr-71 (0.59) nhr-6 nhr-43 | 67 | 1.36 | 3.0e-03 |
| Vsx1\_1728 |  | alr-1 | 55 | 1.52 | 3.3e-03 |
| pTH8863 |  | hmg-12 | 66 | 1.37 | 3.3e-03 |
| Pou2f1\_3081 |  | ceh-18 (0.57) | 31 | 2.07 | 3.4e-03 |
| Egr1\_2580 |  | ZC328.2 | 39 | 1.82 | 3.5e-03 |
| pTH9900 |  | C46E10.8 | 61 | 1.43 | 3.5e-03 |
| pTH5111 |  | mxl-1 aha-1 hlh-30 | 44 | 1.70 | 3.7e-03 |
| Emx2\_3420 |  | ceh-2 | 43 | 1.72 | 3.7e-03 |
| PAX5\_f1 |  | pax-2 (0.51) pax-3 | 42 | 1.73 | 4.1e-03 |
| Lmx1b\_3433 |  | lim-7 lim-6 | 51 | 1.56 | 4.1e-03 |
| Jundm2\_0911 |  | fos-1 | 50 | 1.58 | 4.1e-03 |
| V$CDC5\_01 |  | irx-1 D1081.8 | 65 | 1.37 | 4.2e-03 |
| Zfp652\_1 |  | ZK177.3 | 53 | 1.53 | 4.3e-03 |
| pTH9282 |  | attf-1 C01B12.2 | 34 | 1.93 | 4.5e-03 |
| pTH10722 |  | ref-2 eor-1 egrh-3 | 47 | 1.63 | 4.5e-03 |
| Hoxc6\_3954 |  | pal-1 lin-39 ceh-12 ceh-43 | 62 | 1.40 | 4.6e-03 |
| Osr2\_1727 |  | odd-1 | 69 | 1.31 | 4.7e-03 |
| MA0543.1 |  | eor-1 daf-8 | 62 | 1.40 | 4.7e-03 |
| Cart1\_1275 |  | ceh-18 (0.57) alr-1 lim-7 | 78 | 1.19 | 5.1e-03 |
| Hoxb3\_1720 |  | lin-39 | 74 | 1.25 | 5.3e-03 |
| pTH5914 |  | attf-1 | 29 | 2.09 | 5.4e-03 |
| pTH9219 |  | xbp-1 (0.56) C01B12.2 | 62 | 1.40 | 5.4e-03 |
| pTH9244 |  | tbx-39 | 50 | 1.56 | 5.4e-03 |
| V$ZID\_01 |  | skn-1 ztf-28 | 72 | 1.27 | 5.4e-03 |
| V$MEF2\_04 |  | mef-2 | 59 | 1.43 | 5.5e-03 |
| pTH9237 |  | mel-28 | 56 | 1.47 | 5.5e-03 |
| Hoxd10\_2368 |  | php-3 lin-39 | 64 | 1.37 | 5.6e-03 |
| pTH10031 |  | mbr-1 (0.89) | 55 | 1.48 | 6.0e-03 |
| pTH6478 |  | lim-7 | 45 | 1.64 | 6.2e-03 |
| Elf5 |  | C24A1.2 | 63 | 1.38 | 6.2e-03 |
| pTH6591 |  | lin-31 | 69 | 1.30 | 6.5e-03 |
| Tcf1\_2666 |  | hmbx-1 | 68 | 1.32 | 6.5e-03 |
| Bsx\_3483 |  | ceh-31 | 72 | 1.27 | 6.6e-03 |
| K562\_ZBTB7A\_HudsonAlpha |  | ZC328.2 | 60 | 1.41 | 6.7e-03 |
| PDX1\_1 |  | ceh-31 lin-39 ceh-14 | 79 | 1.17 | 6.9e-03 |
| MA0510.1 |  | daf-19 (0.83) | 68 | 1.31 | 6.9e-03 |
| En1\_3123 |  | ceh-53 ceh-16 | 52 | 1.52 | 7.0e-03 |
| Elf3 |  | C24A1.2 | 80 | 1.15 | 7.1e-03 |
| V$AHR\_01 |  | ahr-1 | 65 | 1.35 | 7.1e-03 |
| retn\_SANGER\_5\_FBgn0004795 |  | ceh-20 cfi-1 let-381 | 55 | 1.47 | 7.3e-03 |
| Elk4 |  | F19F10.1 lin-1 C24A1.2 | 53 | 1.50 | 7.5e-03 |
| pTH9884 |  | tbx-39 | 75 | 1.23 | 7.6e-03 |
| ARNT2\_si |  | aha-1 C46E10.9 | 28 | 2.08 | 7.6e-03 |
| V$GATA3\_01 |  | elt-1 | 70 | 1.29 | 7.7e-03 |
| Hoxb8\_3780 |  | lin-39 | 75 | 1.22 | 7.9e-03 |
| pTH9915 |  | zip-3 | 50 | 1.54 | 7.9e-03 |
| MA0499.1 |  | hlh-1 hlh-11 | 48 | 1.57 | 8.1e-03 |
| MA0473.1 |  | lin-1 C24A1.2 | 33 | 1.89 | 8.3e-03 |
| pTH10015 |  | ces-1 che-1 ztf-28 | 50 | 1.53 | 8.4e-03 |
| pTH9242 |  | mel-28 | 50 | 1.53 | 8.4e-03 |
| HNF4G\_f1 |  | nhr-239 nhr-62 | 69 | 1.29 | 8.4e-03 |
| Spdef |  | lin-1 | 71 | 1.27 | 8.6e-03 |
| pTH1014 |  | atf-5 | 67 | 1.32 | 8.6e-03 |
| Nkx2-6\_3437 |  | ceh-24 (0.59) dsc-1 | 72 | 1.26 | 8.6e-03 |
| pTH2673 |  | fkh-10 let-381 lin-31 | 72 | 1.26 | 8.7e-03 |
| pTH9297 |  | ceh-18 (0.57) | 65 | 1.34 | 8.9e-03 |
| pTH9907 |  | nhr-34 | 77 | 1.19 | 9.0e-03 |
| PLAG1\_si |  | Y53H1A.2 plp-2 | 40 | 1.71 | 9.1e-03 |
| HIF1A\_si |  | hif-1 (0.65) | 64 | 1.35 | 9.2e-03 |
| Tcf7\_0950 |  | pop-1 | 73 | 1.24 | 9.3e-03 |
| Nsy-7 |  | nsy-7 | 42 | 1.66 | 9.5e-03 |
| pTH5887 |  | lin-39 | 68 | 1.30 | 9.6e-03 |
| pTH6268 |  | ceh-2 | 41 | 1.68 | 9.7e-03 |
| Atf1\_3026 |  | crh-1 | 71 | 1.27 | 9.8e-03 |
| Zfp691\_0895 |  | F21A9.2 | 49 | 1.54 | 9.9e-03 |
| pTH10013 |  | nhr-168 | 61 | 1.38 | 9.9e-03 |
| ETS2\_f1 |  | lin-1 C24A1.2 | 35 | 1.81 | 9.9e-03 |
| FLI1\_4 |  | lin-1 | 14 | 3.23 | 1.0e-02 |
| Elf3\_3876 |  | C24A1.2 | 79 | 1.16 | 1.0e-02 |
| TBX2\_2 |  | tbx-39 mab-9 | 75 | 1.22 | 1.0e-02 |
| Barx1\_2877 |  | ceh-43 | 37 | 1.76 | 1.0e-02 |
| V$BRN2\_01 |  | ceh-18 (0.57) | 72 | 1.25 | 1.1e-02 |
| MA0467.1 |  | tbx-39 ceh-45 | 50 | 1.52 | 1.1e-02 |
| Etv3 |  | lin-1 | 76 | 1.20 | 1.1e-02 |
| EMX1\_2 |  | ceh-2 | 39 | 1.71 | 1.1e-02 |
| rn\_SOLEXA\_5\_FBgn0259172 |  | lin-29 | 74 | 1.23 | 1.1e-02 |
| pTH3998 |  | tbx-39 | 50 | 1.51 | 1.1e-02 |
| pTH5924 |  | nhr-255 | 41 | 1.66 | 1.1e-02 |
| Gsh2\_3990 |  | ceh-31 | 45 | 1.59 | 1.2e-02 |
| pTH8318 |  | attf-1 | 59 | 1.39 | 1.2e-02 |
| pTH3811 |  | nhr-2 nhr-213 nhr-19 nhr-69 | 63 | 1.35 | 1.2e-02 |
| RFX5\_2 |  | daf-19 (0.83) F52B5.7 | 65 | 1.33 | 1.2e-02 |
| MA0531.1 |  | Y5F2A.4 | 39 | 1.70 | 1.2e-02 |
| pTH7876 |  | mel-28 lin-29 | 76 | 1.20 | 1.2e-02 |
| MA0249.1 |  | hlh-8 | 46 | 1.57 | 1.2e-02 |
| pTH5539 |  | unc-120 | 72 | 1.25 | 1.2e-02 |
| Hoxb4\_2627 |  | lin-39 | 75 | 1.21 | 1.3e-02 |
| Barhl1\_1 |  | ceh-31 | 72 | 1.25 | 1.3e-02 |
| IRX5\_1 |  | irx-1 | 34 | 1.81 | 1.3e-02 |
| pTH10768 |  | med-2 (0.71) | 65 | 1.32 | 1.3e-02 |
| Oli\_da\_SANGER\_5\_3\_FBgn0032651 |  | hlh-32 | 71 | 1.26 | 1.4e-02 |
| dsx-F\_FlyReg\_FBgn0000504 |  | sox-4 (0.74) grh-1 dmd-4 | 58 | 1.40 | 1.4e-02 |
| pTH6106 |  | nhr-182 | 42 | 1.63 | 1.4e-02 |
| ZN384\_f1 |  | K11D2.4 lin-29 | 60 | 1.37 | 1.4e-02 |
| pTH9384 |  | cfi-1 | 49 | 1.51 | 1.4e-02 |
| pTH9256 |  | ceh-18 (0.57) | 65 | 1.32 | 1.4e-02 |
| V$CEBP\_01 |  | C48E7.11 | 63 | 1.34 | 1.5e-02 |
| SRP001585\_Tbx2 |  | odr-7 tbx-39 | 74 | 1.22 | 1.5e-02 |
| Spdef\_0905 |  | lin-1 | 43 | 1.60 | 1.5e-02 |
| Irx3\_2226 |  | irx-1 | 70 | 1.26 | 1.5e-02 |
| pTH2846 |  | lin-31 | 65 | 1.31 | 1.6e-02 |
| Hoxb5\_3122 |  | lin-39 | 28 | 1.97 | 1.6e-02 |
| SOX10\_1 |  | sox-4 (0.74) | 71 | 1.25 | 1.6e-02 |
| pTH6641 |  | lin-31 | 67 | 1.29 | 1.6e-02 |
| TCF7L1\_1 |  | pop-1 | 65 | 1.31 | 1.6e-02 |
| pTH5561 |  | nhr-239 | 71 | 1.25 | 1.6e-02 |
| pTH2283 |  | odd-2 | 58 | 1.39 | 1.7e-02 |
| pTH9925 |  | ztf-11 | 52 | 1.46 | 1.7e-02 |
| Vax1\_3499 |  | C02F12.10 | 37 | 1.71 | 1.7e-02 |
| FOXG1\_2 |  | lin-31 | 25 | 2.08 | 1.7e-02 |
| pTH8556 |  | pax-2 (0.51) | 32 | 1.83 | 1.7e-02 |
| I$UBX\_01 |  | lin-39 | 67 | 1.29 | 1.8e-02 |
| HXD13\_f1 |  | pal-1 | 64 | 1.32 | 1.8e-02 |
| pTH8998 |  | mab-3 | 56 | 1.41 | 1.8e-02 |
| MA0386.1 |  | che-1 K11D2.4 tbp-1 | 79 | 1.15 | 1.8e-02 |
| MA0117.1 |  | F45H11.6 | 67 | 1.29 | 1.8e-02 |
| pTH9222 |  | mel-28 | 50 | 1.48 | 1.9e-02 |
| pTH9082 |  | mab-23 | 63 | 1.33 | 1.9e-02 |
| pTH10624 |  | Y61A9LA.9 | 66 | 1.30 | 1.9e-02 |
| pTH9177 |  | F10B5.3 (0.67) Y53C10A.3 | 60 | 1.36 | 1.9e-02 |
| MA0126.1 |  | pax-3 lin-48 | 49 | 1.49 | 2.0e-02 |
| pTH7875 |  | mel-28 | 44 | 1.56 | 2.0e-02 |
| pTH9108 |  | daf-12 | 74 | 1.21 | 2.1e-02 |
| Mw142 |  | egl-27 | 61 | 1.35 | 2.1e-02 |
| pTH10721 |  | ztf-9 (0.52) | 41 | 1.61 | 2.1e-02 |
| pTH3510 |  | nhr-86 | 63 | 1.32 | 2.1e-02 |
| pTH9262 |  | lin-54 | 63 | 1.32 | 2.1e-02 |
| VDR\_1 |  | gei-3 (0.54) nhr-208 | 47 | 1.51 | 2.1e-02 |
| V$MYB\_Q6 |  | D1081.8 | 42 | 1.59 | 2.1e-02 |
| Pou3f4\_3773 |  | ceh-6 | 61 | 1.34 | 2.2e-02 |
| Nkx6-3\_3446 |  | cog-1 | 56 | 1.40 | 2.2e-02 |
| ARNT\_f1 |  | hlh-27 aha-1 | 63 | 1.32 | 2.3e-02 |
| pTH5082 |  | atf-2 | 48 | 1.49 | 2.3e-02 |
| HXC6\_f1 |  | nhr-100 (0.61) lin-39 lin-1 | 60 | 1.35 | 2.3e-02 |
| Hoxa4\_3426 |  | lin-39 | 74 | 1.21 | 2.3e-02 |
| Elf4 |  | C24A1.2 | 56 | 1.39 | 2.4e-02 |
| Zbtb12\_2932 |  | lsy-27 | 20 | 2.29 | 2.4e-02 |
| Hoxc4\_3491 |  | lin-39 | 74 | 1.20 | 2.5e-02 |
| Pknox2\_3077 |  | ceh-32 | 69 | 1.25 | 2.5e-02 |
| pTH9096 |  | T07C12.11 | 18 | 2.42 | 2.6e-02 |
| MA0456.1 |  | ref-2 | 35 | 1.71 | 2.6e-02 |
| pTH10633 |  | R07H5.10 C48E7.11 | 54 | 1.41 | 2.6e-02 |
| pTH5078 |  | ces-2 | 41 | 1.59 | 2.7e-02 |
| Nkx6-1\_2825 |  | cog-1 | 24 | 2.05 | 2.7e-02 |
| pTH3477 |  | daf-16 | 62 | 1.32 | 2.7e-02 |
| DLX2\_f1 |  | lin-39 hmg-12 ceh-53 lin-31 ceh-43 | 75 | 1.19 | 2.8e-02 |
| Vax2\_3500 |  | C02F12.10 | 36 | 1.68 | 2.8e-02 |
| pTH10038 |  | sox-4 (0.74) gei-3 (0.54) | 62 | 1.32 | 2.8e-02 |
| pTH9924 |  | nhr-46 (0.53) | 49 | 1.47 | 2.8e-02 |
| Nkx1-1\_3856 |  | ceh-30 | 26 | 1.95 | 2.8e-02 |
| pTH9026 |  | attf-1 | 56 | 1.38 | 2.8e-02 |
| V$FAC1\_01 |  | gei-8 | 73 | 1.21 | 2.9e-02 |
| pTH5976 |  | mxl-1 irx-1 | 44 | 1.53 | 2.9e-02 |
| pTH10629 |  | ztf-3 | 32 | 1.77 | 2.9e-02 |
| V$S8\_01 |  | ceh-45 | 78 | 1.15 | 2.9e-02 |
| pTH10041 |  | ztf-29 | 47 | 1.49 | 2.9e-02 |
| Hoxa10\_2318 |  | ceh-24 (0.59) | 42 | 1.56 | 2.9e-02 |
| ZNF75A\_1 |  | lag-1 ztf-3 | 65 | 1.29 | 3.0e-02 |
| pTH8216 |  | Y116A8C.22 | 19 | 2.31 | 3.0e-02 |
| Hoxa3\_2783 |  | lin-39 | 71 | 1.23 | 3.0e-02 |
| Hoxa11\_2218 |  | php-3 | 55 | 1.39 | 3.0e-02 |
| pTH6562 |  | ceh-5 | 39 | 1.61 | 3.0e-02 |
| Irx5\_2385 |  | irx-1 | 69 | 1.25 | 3.0e-02 |
| GRHL1\_1 |  | grh-1 | 58 | 1.36 | 3.1e-02 |
| pTH10630 |  | lsy-27 | 32 | 1.76 | 3.1e-02 |
| pTH9043 |  | sem-2 | 57 | 1.37 | 3.1e-02 |
| pTH9279 |  | Y116A8C.22 | 52 | 1.42 | 3.4e-02 |
| pTH8985 |  | athp-1 | 69 | 1.24 | 3.4e-02 |
| pTH6436 |  | ceh-53 | 40 | 1.58 | 3.5e-02 |
| pTH3751 |  | tbx-39 | 50 | 1.44 | 3.5e-02 |
| Mafk\_3106 |  | F45H11.6 | 48 | 1.46 | 3.5e-02 |
| pTH6327 |  | dsc-1 | 70 | 1.23 | 3.6e-02 |
| Hlxb9\_3422 |  | ceh-12 | 71 | 1.22 | 3.6e-02 |
| pTH9245 |  | ceh-18 (0.57) | 34 | 1.69 | 3.6e-02 |
| pTH6486 |  | nhr-145 | 52 | 1.41 | 3.7e-02 |
| pTH9163 |  | nhr-3 (0.53) | 58 | 1.35 | 3.8e-02 |
| MA0095.2 |  | lsy-2 | 61 | 1.32 | 3.8e-02 |
| PTF1A\_f1 |  | lin-32 | 74 | 1.19 | 3.8e-02 |
| GATA6\_f2 |  | elt-1 | 43 | 1.52 | 3.8e-02 |
| Eip93F\_SANGER\_10\_FBgn0013948 |  | mbr-1 (0.89) F26F4.8 | 71 | 1.22 | 3.9e-02 |
| pTH5119 |  | cfi-1 | 39 | 1.59 | 3.9e-02 |
| pTH5922 |  | ceh-24 (0.59) | 35 | 1.66 | 3.9e-02 |
| Pou3f3\_3235 |  | ceh-6 | 75 | 1.18 | 3.9e-02 |
| Hoxa5\_3415 |  | lin-39 | 27 | 1.86 | 4.0e-02 |
| pTH5714 |  | nhr-239 | 46 | 1.48 | 4.0e-02 |
| MA0505.1 |  | nhr-68 | 67 | 1.26 | 4.0e-02 |
| pTH5267 |  | hlh-32 | 17 | 2.38 | 4.0e-02 |
| Evx1\_3952 |  | ceh-53 | 26 | 1.89 | 4.1e-02 |
| pTH9182 |  | tbx-39 | 47 | 1.46 | 4.1e-02 |
| GATA5\_f1 |  | elt-1 | 62 | 1.30 | 4.1e-02 |
| V$IK2\_01 |  | F26F4.8 | 71 | 1.22 | 4.2e-02 |
| PAX8\_f1 |  | pax-2 (0.51) | 40 | 1.56 | 4.2e-02 |
| Hoxa7\_2668 |  | lin-39 | 72 | 1.21 | 4.2e-02 |
| pTH9300 |  | dmd-3 | 43 | 1.51 | 4.4e-02 |
| V$HOX13\_01 |  | lin-39 | 35 | 1.65 | 4.4e-02 |
| pTH10772 |  | ceh-52 | 39 | 1.57 | 4.7e-02 |
| PO3F2\_si |  | ceh-18 (0.57) | 74 | 1.18 | 4.7e-02 |
| Hmx2\_3424 |  | ceh-9 | 75 | 1.17 | 4.9e-02 |

### Correlated (and anti-correlated) transcription factors

|  |  |
| --- | --- |
| **Transcription factor** | **Correlation** |
| mbr-1 | 0.89 |
| ceh-62 | 0.86 |
| ctbp-1 | 0.84 |
| tag-97 | 0.84 |
| daf-3 | 0.84 |
| nhr-158 | 0.83 |
| daf-19 | 0.83 |
| nhr-1 | 0.81 |
| nhr-51 | 0.81 |
| lfi-1 | 0.80 |
| nhr-277 | 0.79 |
| nhr-52 | 0.79 |
| egl-13 | 0.77 |
| ttx-3 | 0.74 |
| nhr-4 | 0.74 |
| ceh-58 | 0.74 |
| sox-4 | 0.74 |
| nhr-17 | 0.74 |
| nhr-124 | 0.73 |
| madf-4 | 0.72 |
| ets-5 | 0.72 |
| nhr-258 | 0.72 |
| med-2 | 0.71 |
| C34F6.9 | 0.71 |
| crh-2 | 0.71 |
| vab-15 | -0.42 |
| lst-5 | -0.42 |
| nhr-111 | -0.42 |
| nhr-256 | -0.43 |
| C30G4.7 | -0.44 |
| sma-4 | -0.44 |
| ztf-13 | -0.45 |
| pal-1 | -0.45 |
| ceh-82 | -0.45 |
| nob-1 | -0.45 |
| nhr-261 | -0.45 |
| zip-7 | -0.45 |
| cep-1 | -0.46 |
| ces-1 | -0.46 |
| sup-35 | -0.47 |
| nhr-9 | -0.47 |
| hlh-29 | -0.47 |
| Y48G9A.11 | -0.48 |
| ceh-40 | -0.48 |
| dmd-3 | -0.50 |
| F27D4.4 | -0.51 |
| nhr-210 | -0.51 |
| Y56A3A.18 | -0.52 |
| tbx-41 | -0.53 |
| nhr-13 | -0.58 |

### ChIP peaks enriched

|  |  |  |  |  |
| --- | --- | --- | --- | --- |
| **Gene** | **Experiment** | **Number of upstream peaks** | **Enrichment** | **FDR corrected p** |
| C34F6.9 | C34F6.9\_Larvae-L2-stage | 18 | 2.45 | 0.011 |
